# The Impact of Chronic Severe Preoperative Local Infection on Postoperative Complications and Recurrence Following Winograd Procedure for Ingrown Toenail of the Great Toes: A Retrospective Cohort Study

**DOI:** 10.3390/jcm15176577

**Published:** 2026-08-26

**Authors:** Ji Yong Yun, Jin Soo Suh, Yun Beom Lee, Jun Young Choi

**Affiliations:** Department of Orthopedic Surgery, Inje University Ilsan Paik Hospital, 170 Juhwa-ro, Ilsanseo-gu, Goyang 10380, Gyeonggi-do, Republic of Korea; i8170@paik.ac.kr (J.Y.Y.); sjs0506@paik.ac.kr (J.S.S.); i8126@paik.ac.kr (Y.B.L.)

**Keywords:** ingrown toenail, ingrowing toenail, Winograd procedure, preoperative infection, recurrence, antibiotics

## Abstract

**Background/Objectives**: The Winograd procedure is a widely utilized surgical option for treating ingrown toenails. However, there is a lack of comparative data regarding its outcomes in the presence of active infection. The objective of this study was to compare the clinical outcomes and complication rates of the Winograd procedure for great toe ingrown toenails between patients with and without chronic severe preoperative local infection. **Methods**: This retrospective study included 388 great toes that underwent the Winograd procedure for ingrown toenails between 2015 and 2025 with minimum 6 months’ follow-up. Cases were categorized into two groups based on the presence (Group 1, *n* = 76) or absence (Group 2, *n* = 312) of preoperative signs of severe local infection (abscess, granulation tissue formation, and/or foul odor). Demographic factors, and clinical outcomes, including postoperative infection rates, antibiotic duration, and recurrence rates, were evaluated and compared between the two groups. **Results**: Patients in Group 1 were significantly younger (29.7 vs. 39.5 years; *p* = 0.001) and had a higher smoking prevalence (11.8% vs. 5.1%; *p* = 0.033) than those in Group 2. Postoperatively, the mean duration of antibiotic administration (13.9 vs. 10.6 days; *p* = 0.114) and the incidence of superficial operative site infection (31.6% vs. 28.2%; *p* = 0.561) showed no significant differences between the two groups. Persistent infection lasting more than three weeks was comparable (15.8% vs. 11.5%; *p* = 0.313), with similar antibiotic durations in these cases (33.0 vs. 36.6 days; *p* = 0.094). Most importantly, the postoperative recurrence rate did not differ significantly between the two groups, with 13.2% in Group 1 compared to 15.3% in Group 2 (*p* = 0.625). **Conclusions**: Immediate Winograd procedure with radical excision of infected granulation tissue showed no statistically significant differences in postoperative complication or recurrence rates, even in patients with chronic severe preoperative local infection. These findings suggest that immediate surgical intervention can be considered a feasible treatment option for direct source removal.

## 1. Introduction

Ingrown toenail, or onychocryptosis, is a prevalent foot disorder characterized by the nail plate encroaching into the surrounding soft tissues, leading to significant pain and inflammation [1]. Its etiology is multifactorial, involving a complex interplay of improper nail trimming, abnormal nail and nail-fold morphology, hyperhidrosis, ill-fitting footwear, poor hygiene, obesity, genetic predisposition, trauma, fungal infections, and various anatomical factors [2]. Clinically, as the nail plate penetrates the periungual soft tissue, patients present with a spectrum of symptoms, including excruciating pain, swelling, discharge, abscess formation, and hypertrophic granulation tissue [3,4]. Among these, stabbing pain and functional discomfort are the most common complaints [5], predominantly affecting adolescents and young adults, thereby significantly impairing their daily and occupational activities [2]. If left untreated, the condition frequently progresses to a chronic inflammatory state with recurrent local infections, resulting in permanent nail deformity and persistent symptomatic distress [6,7].

While various surgical interventions have been described, the Winograd procedure [8] remains one of the most widely utilized techniques [3]. This procedure involves a partial nail plate avulsion combined with a formal excision of the germinal matrix (matricectomy), followed by primary closure of the soft tissue [4,5]. It is recognized as an effective treatment due to its relatively low recurrence rates and high patient satisfaction, and its suitability for outpatient settings minimizes postoperative recovery time [4,5].

Despite its popularity, there is a distinct lack of literature directly comparing outcomes of the Winograd procedure specifically in the presence versus absence of active, severe local infection. Given that ingrown toenails frequently present with secondary paronychia or soft tissue infection, surgeons often face clinical uncertainty regarding whether to delay surgery for infection control or proceed with immediate source removal. To address this clinical uncertainty, the primary objective of this study was to compare the postoperative clinical course and complication rates of the Winograd procedure for ingrown toenails of the great toes between patients with and without chronic severe preoperative local infection. Based on the traditional rationale regarding surgical site contamination, we explicitly hypothesized that chronic severe preoperative local infection would be associated with an increased incidence of postoperative surgical site infection, a prolonged duration of antibiotic therapy, and higher recurrence rates.

## 2. Materials and Methods

### 2.1. Patient Selection

A retrospective cohort design was adopted to efficiently analyze a large single-center sample over a 10-year period while avoiding the ethical and logistical constraints of prospectively randomizing or delaying surgical intervention in patients presenting with severe, painful infections. We retrospectively identified consecutive patients from our institutional surgical database who underwent the Winograd procedure for ingrown toenails of the great toe (ICD-10 code L60.0) during a 10-year period from March 2015 to March 2025. (The study data collection and analysis were conducted in April 2026 following institutional protocols.)

Patients were included if they underwent the Winograd procedure for great toe ingrown toenails and completed a minimum clinical follow-up of 6 months, evaluated either through routine chart review or standardized telephone recall. The exclusion criteria were as follows: (1) radiographic or clinical evidence of osteomyelitis progression, and (2) a history of prior surgery at the same site. Patients lost to follow-up due to a lack of response during telephone recall were also excluded from the final analysis. Eligible cases were categorized into two groups based on the clinical status of the affected great toe at the time of surgery: Group 1 (chronic severe preoperative local infection) and Group 2 (no or mild preoperative local infection). In this study, “chronic” was operationally defined as symptomatic local infection persisting or recurring for at least 4 weeks despite conservative treatment. “Severe” was operationally defined as the presence of advanced infectious physical signs, including exuberant hypertrophic granulation tissue formation, overt purulent discharge, or localized. Conversely, Group 2 comprised patients with no or mild preoperative infection presenting with localized erythema, edema, or focal tenderness without prominent tissue hyper-trophy or purulent discharge.

### 2.2. Sample Size Calculation

To ensure adequate statistical power for comparing the two groups, the required sample size was calculated based on the primary endpoint: the postoperative recurrence rate. Based on previously reported data regarding the Winograd procedure, we assumed a baseline recurrence rate of approximately 12% [9]. To detect a 15% difference in recurrence rates between the chronic severe preoperative local infection and non-severe infection groups with a two-sided significance level (α) of 0.05 and a statistical power (1 − β) of 80%, a minimum of 68 patients per group was required. Given that our final analysis included 76 great toes in the chronic severe local infection group and 312 in the no or mild local infection group, the sample size was deemed sufficient to provide adequate power for the statistical comparisons.

### 2.3. Operative Technique

**Figure 1:**
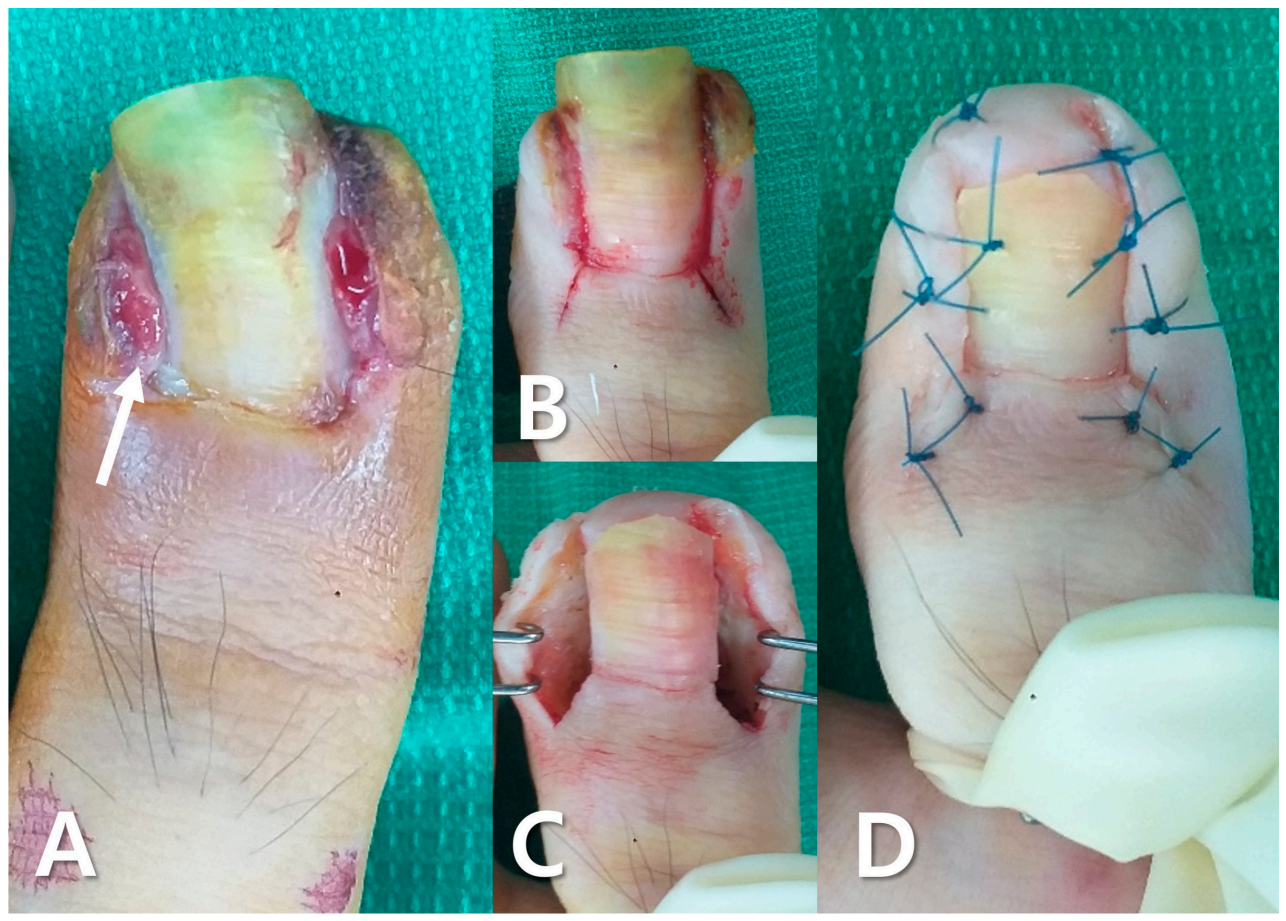
Surgical procedure for an ingrown toenail with chronic severe preoperative local infection. Preoperative clinical photograph showing an ingrown toenail of the great toe with signs of infection and associated granulation tissue (arrow, (**A**)). A longitudinal incision is made through the nail plate and overlying eponychium, followed by thorough debridement to excise all inflammatory and hypertrophic tissues (**B**). Wedge resection is performed, encompassing the affected nail portion and the underlying matrix. The soft tissue is meticulously excised until the periosteum of the distal phalanx is clearly visualized to ensure complete matricectomy (**C**). Postoperative view after suturing the lateral nail fold to the nail plate with non-absorbable sutures to obliterate the dead space (**D**).

All surgeries were performed by two fellowship-trained foot and ankle surgeons (JYC, JSS) under local digital nerve blocks. A tourniquet was applied at the base of the great toe to maintain a bloodless surgical field. First, a longitudinal incision was made through the nail plate and the overlying eponychium, extending proximally to the germinal matrix (Figure 1A,B). In patients with chronic severe local infection associated with granulation tissue, a thorough debridement was performed to excise all inflammatory and hypertrophic tissues (Figure 1B). A wedge resection was then executed, encompassing the affected nail portion, the underlying sterile and germinal matrix (Figure 1C). During the matricectomy, the soft tissue was meticulously excised until the periosteum of the distal phalanx was clearly visualized to ensure complete removal of the germinal cells. To further minimize the risk of recurrence, the remaining germinal matrix and nail bed underwent thorough electrocauterization. After ensuring hemostasis, the remaining soft tissue of the lateral nail fold was sutured to the nail plate using non-absorbable sutures to obliterate the dead space (Figure 1D). A sterile compression dressing was applied. Postoperatively, a standardized oral antibiotic regimen consisting of Cefatrizine (Cephamethyl^®^; a first-generation cephalosporin, 500 mg per tablet) was routinely prescribed at a dosage of 1000 mg (two tablets) three times daily (total 3000 mg/day) for 5 to 7 days. In cases where superficial surgical site infection persisted during follow-up, the duration of antibiotic administration was extended until clinical eradication was achieved. Wound cultures were not routinely performed given the outpatient setting; however, in select cases with overt abscess formation where cultures were obtained, Staphylococcus aureus was the most frequently isolated pathogen, all of which responded well to the primary empirical cephalosporin regimen. All sutures were removed 3 weeks after the surgery once the wound had sufficiently healed.

### 2.4. Assessments

Patient demographic data were extracted from the institutional electronic medical records, including age at the time of surgery, sex, smoking status at the time of surgery, and past medical history such as hypertension, diabetes mellitus, coronary heart disease, end-stage renal disease, and thyroid diseases. Regarding the surgical site, the affected side (right or left) and the specific location of the ingrown toenail (medial, lateral, or both) were documented for each great toe. The follow-up duration was recorded for all included cases to ensure a minimum clinical tracking period. The primary clinical focus was the comparison of the postoperative course between Group 1 and 2. Therefore, the following parameters were evaluated and analyzed: the mean duration of oral antibiotic administration after the surgery, the occurrence of superficial surgical site infections (defined as an infection occurring within 30 days postoperatively involving only the skin and subcutaneous tissue, accompanied by purulent drainage or localized signs such as pain, erythema, and warmth), and cases of “persistent surgical site infection” defined as an infection lasting more than three weeks postoperatively. In these persistent cases, the total duration of antibiotic use was further quantified.

Additionally, postoperative recurrence, defined as the regrowth of the nail spicule or the return of ingrown symptoms requiring further intervention, was documented. Regarding cases followed up via telephone recall, a structured interview was conducted to assess recurrence and complications. Patients were specifically asked about the presence of purulent discharge, the need for prolonged antibiotic use, and the regrowth of any nail spicules. To ensure data integrity and minimize investigator bias, all clinical data and outcomes were independently extracted and reviewed by two orthopedic researchers (JYY, YBL) using predefined operational definitions. In cases of disagreement regarding outcome categorization or complication presence, final decisions were reached through joint case review and consensus with a senior attending orthopedic surgeon (JSS).

### 2.5. Statistical Analyses

Statistical analyses were conducted using SPSS software (version 21.0; IBM Corp., Armonk, NY, USA). The normality of data distribution was assessed using the Shapiro–Wilk test. Continuous numeric variables between the two groups were compared using the independent *t*-test, whereas categorical variables and proportions were analyzed using the chi-square test. To control for baseline discrepancies between the groups, multivariable logistic regression analysis was performed. Confounding variables included in the model (age and smoking status) were selected based on baseline clinical relevance and statistically significant differences observed between Group 1 and Group 2 in univariate comparisons (p<0.05). Adjusted odds ratios (aORs) and 95% confidence intervals (CIs) were determined for each outcome. Additionally, to evaluate the robustness of our findings against potential recall bias from telephone follow-up, a sensitivity analysis was performed restricting the multivariable logistic regression to patients whose outcomes were documented exclusively through in-person chart reviews (n=209). Missing data resulting from loss to follow-up during telephone recall (78 cases, 16.7%) were managed using complete case analysis, with these incomplete cases excluded from the final analytical dataset. For the remaining 388 included great toes, all demographic and clinical variable records were complete, with no missing outcome or covariate data requiring statistical imputation. For all comparisons, statistical significance was defined as *p* < 0.05.

## 3. Results

### 3.1. Patient Flow and Selection

**Figure 2:**
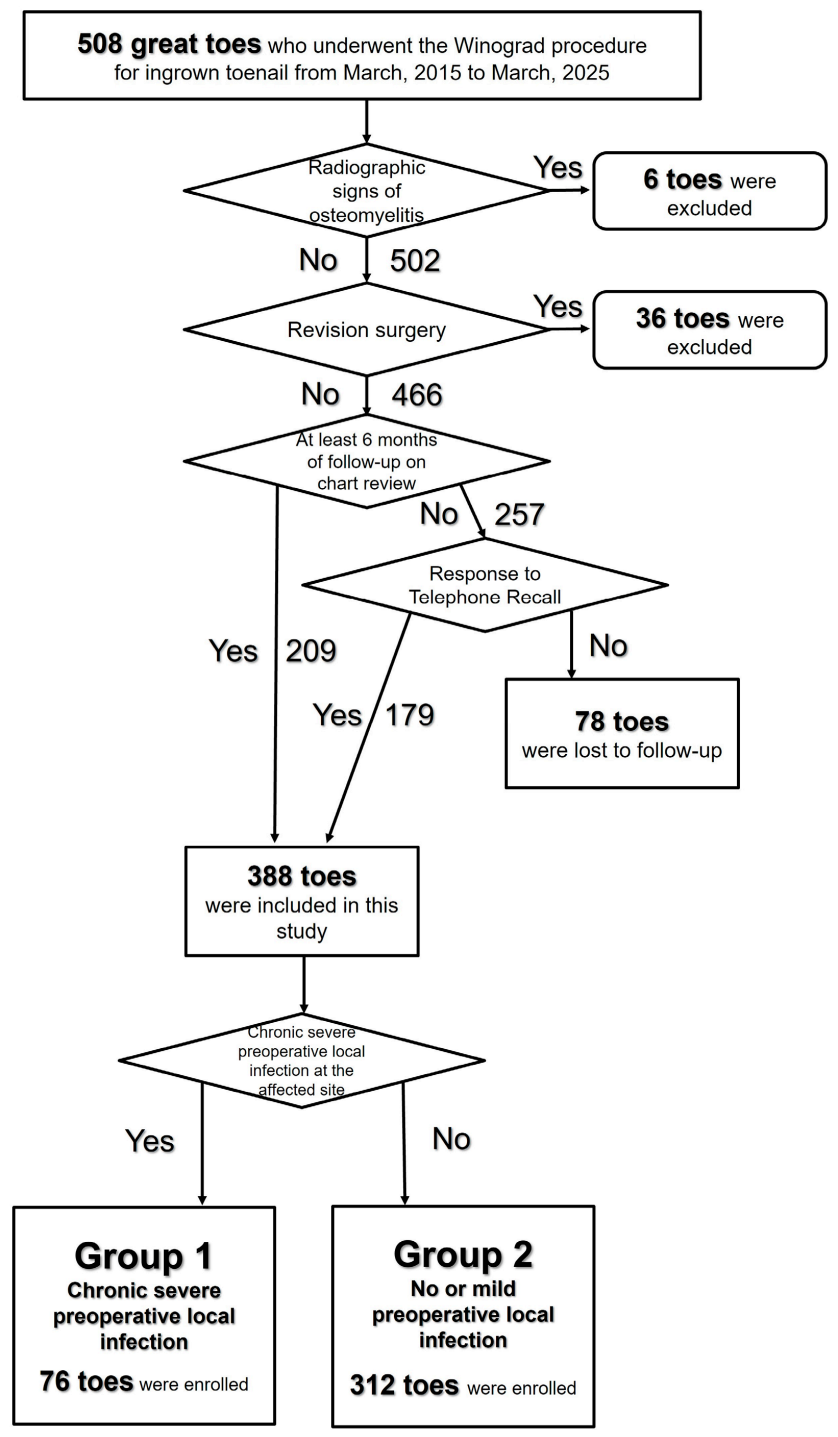
Patient selection algorithm.

Initially, 508 great toes were identified. After a preliminary chart review, 6 toes were excluded due to radiographic signs or clinical records indicating progression to osteomyelitis, and 36 toes were excluded due to a history of revision surgery at the same site. Of the remaining 466 great toes, we prioritized those with a minimum clinical follow-up of 6 months. While 209 toes met this criterion through routine chart review, the remaining 257 cases were contacted via telephone recall to evaluate their long-term status. Among these, 179 toes were successfully followed up, while 78 toes were lost to follow-up due to a lack of response. Consequently, a total of 388 great toes were included in the final analysis. These cases were further categorized into two groups based on the clinical status of the affected great toe at the time of surgery: Group 1, 76 great toes with preoperative signs of chronic severe local infection (exuberant granulation tissue formation, and/or abscess); and Group 2, 312 great toes with no or mild signs of preoperative local infection (erythema, edema, and focal tenderness). A detailed patient selection algorithm is illustrated in Figure 2.

A total of 388 great toes were included in this study. The cases were categorized into two groups based on the presence or absence of chronic severe preoperative local infection at the affected site: 76 great toes with chronic severe preoperative signs of local infection and 312 great toes with no or mild preoperative local infection.

### 3.2. Overall Patient Characteristics

The mean age of the patients at the time of surgery was 37.6 ± 20.3 years (range, 10–89 years). There were 149 great toes (38.4%) from female patients. Smoking at the time of surgery was reported in 25 cases (6.4%). Regarding past medical history, the most common comorbidities were hypertension (10.2%) and diabetes mellitus (8.9%), followed by coronary heart disease (3.2%) and end-stage renal disease (1.9%). The affected side was nearly equally distributed between the right (49.2%) and left (50.8%). The ingrown nail was located on the lateral side only in 154 great toes (39.9%), on the medial side only in 128 (32.8%), and on both sides in 106 (27.3%). Preoperative signs of chronic severe local infection at the affected site were observed in 76 great toes (19.6%). The mean follow-up duration was 34.8 ± 6.9 months (range, 6–97 months), ensuring a minimum follow-up of 6 months for all included cases. The overall patient characteristics are summarized in Table 1.

### 3.3. Comparison of Baseline Demographics Between Groups with and Without Chronic Severe Preoperative Local Infection

A comparison of the baseline characteristics between the two groups revealed several significant differences (Table 1). The mean age at the time of surgery was significantly lower in Group 1 compared to Group 2 (29.7 ± 18.5 vs. 39.5 ± 22.7 years; *p* = 0.001). Additionally, the prevalence of smoking at the time of surgery was significantly higher in Group 1 than in Group 2 (11.8% vs. 5.1%; *p* = 0.033). There were no statistically significant differences between the two groups in terms of sex distribution (*p* = 0.173), comorbidities such as hypertension (*p* = 0.262) and diabetes (*p* = 0.098), or the proportion of cases with involvement of both medial and lateral sides (*p* = 0.073). The mean follow-up duration was comparable between the two groups (*p* = 0.125).

### 3.4. Comparison of Postoperative Progress Between Groups with and Without Chronic Severe Preoperative Local Infection

The clinical outcomes and complication rates were analyzed and compared based on the presence of preoperative signs of chronic severe local infection (Table 2). The mean duration of oral antibiotic administration after surgery was 13.9 ± 7.5 days (range, 5–37 days) in Group 1 and 10.6 ± 7.8 days (range, 5–46 days) in Group 2, showing no statistically significant difference (*p* = 0.114). Regarding postoperative complications, superficial surgical site infection occurred in 24 cases (31.6%) of Group 1 and in 88 cases (28.2%) of Group 2, which was not significantly different (*p* = 0.561). Furthermore, the incidence of persistent surgical site infection lasting more than three weeks was comparable between the two groups, observed in 12 (15.8%, Group 1) and 36 (11.5%, Group 2) great toes, respectively (*p* = 0.313). For these persistent cases, the mean duration of antibiotic use was 33.0 ± 3.4 days in Group 1 and 36.6 ± 5.6 days in Group 2 (*p* = 0.094). In all cases, the initial antibiotic regimen—first-generation cephalosporins—was maintained without the need for modification, and no cases required secondary surgical intervention due to a failure of infection eradication. Most importantly, the postoperative recurrence rate did not differ significantly between the groups. Recurrence was observed in 10 cases of Group 1 (13.2%) and in 48 cases of Group 2 (15.3%, *p* = 0.625).

To control for baseline discrepancies between the two groups, multivariable logistic regression analysis was performed, adjusting for age and smoking status. The analysis demonstrated that chronic severe preoperative local infection was not an independent predictor of superficial surgical site infection (aOR = 1.15, 95% CI = 0.68–1.95, *p* = 0.582), persistent infection (aOR = 1.32, 95% CI = 0.62–2.80, *p* = 0.461), or postoperative recurrence (aOR = 0.88, 95% CI = 0.42–1.85, *p* = 0.742). Age and smoking status also showed no significant independent associations with any of the clinical outcomes (Table 3).

In the sensitivity analysis restricted to patients followed up via direct chart review (*n* = 209), chronic severe preoperative local infection similarly demonstrated no significant association with superficial surgical site infection (aOR = 1.12, 95% CI = 0.58–2.15, *p* = 0.731), persistent infection (aOR = 1.25, 95% CI = 0.49–3.18, *p* = 0.640), or postoperative recurrence (aOR = 0.82, 95% CI = 0.31–2.14, *p* = 0.689), confirming the robustness of the primary findings.

## 4. Discussion

Our data suggested that chronic severe preoperative local infection does not negatively impact the clinical outcomes of the Winograd procedure for ingrown toenails. Although patients with chronic severe preoperative local infection were significantly younger and had a higher prevalence of smoking compared to those without it, there were no statistically significant differences between the two groups regarding postoperative complication rates, duration of antibiotic use, or recurrence rates. Specifically, the recurrence rate was 13.2% in the group with chronic severe preoperative local infection and 15.3% in the group with no or mild infection. Furthermore, the incidence of superficial surgical site infection and persistent infection lasting more than three weeks was comparable regardless of the chronic severe preoperative local infection. These findings contrasted with our initial hypothesis, which assumed that chronic severe preoperative local infection would lead to a higher incidence of postoperative complications and recurrence. Instead, our results imply that immediate surgical intervention—focusing on the radical excision of infected and hypertrophic granulation tissue alongside the offending nail spicule—is still an effective strategy.

To assess the severity of ingrown toenails and to guide therapeutic decision-making, the Heifetz classification is widely utilized [10,11,12]. This system categorizes the disease into three distinct stages: Stage 1 is characterized by mild erythema and edema; Stage 2 involves increased inflammation with associated infection; and Stage 3 is defined by chronic inflammation with the formation of exuberant granulation tissue and hypertrophy of the nail fold. Surgical intervention is generally recommended for Stage 2 and Stage 3 cases that prove refractory to conservative management. In our study, the group with severe preoperative local infection—characterized by purulent discharge and significant granulation tissue—corresponds to Heifetz Stage 3. Conversely, cases with no or mild preoperative infection primarily encompassed patients in Stage 1 or Stage 2, where acute inflammatory symptoms remained mild to moderate. By demonstrating that outcomes in Stage 3 cases (Group 1) were comparable to those in earlier stages (Group 2), our results further validate the efficacy of the Winograd procedure across the full spectrum of advanced ingrown toenail severity.

The traditional Winograd procedure [8], which involves a wedge resection of the nail plate and nail bed followed by primary closure, is recognized for its simplicity, safety, and rapid recovery [13]. However, concerns regarding potential cosmetic deformities and reported recurrence rates of approximately 12% have typically limited its application to mild-to-moderate cases [13]. To address these limitations, various modifications have been proposed, such as the modified Winograd technique using curettage and electrocautery to minimize soft tissue excision while precisely removing the matrix [4,14,15]. In our practice, we also incorporated electrocauterization of the remaining nail bed to further minimize recurrence risk. Moreover, we performed wedge resection in all cases regardless of the presence of chronic severe local infection. For severely infected cases, a more extensive debridement of inflammatory and hypertrophic tissues was performed as necessary. Despite our more radical debridement in infected cases, primary wound closure was consistently achievable due to the relatively abundant soft tissue surrounding the great toe. Our results showed an overall recurrence rate of approximately 15%, which is similar to the outcomes reported in existing literature. These findings suggest that the Winograd procedure, when augmented with thorough debridement and electrocautery, remains a viable option even for chronic advanced cases, obviating the need for more morbid, aggressive procedures.

The primary motivation for this study was to determine whether performing immediate surgery on an ingrown toenail with chronic severe local infection increases postoperative complication rates or adversely affects clinical progress. When managing such cases, two surgical strategies can be considered: a delayed Winograd procedure after initial infection control, or an immediate procedure that includes the excision of infected tissues. While the former may allow for a more precise resection in a less inflamed environment, it inevitably prolongs the total treatment duration. Furthermore, as long as the offending nail plate continues to penetrate the soft tissue, the structural cause of the infection remains, which can lead to limited infection control or frequent exacerbations. In contrast, an immediate surgical approach allows for the concurrent removal of the nail plate and germinal matrix, thereby directly addressing the underlying cause of the infection. Potentially facilitating shorter overall treatment periods and more rapid symptomatic relief, such a strategy shares a conceptual parallel with the fundamental surgical principles applied to other musculoskeletal infections, such as septic arthritis, soft tissue abscesses, and chronic osteomyelitis [16,17,18,19]. In these conditions, as long as the infectious focus persists, the inflammatory response will be maintained. Therefore, the radical and prompt removal of the infection source is considered a more critical therapeutic principle than relying solely on antibiotic therapy for infection control. Our findings support the application of this “source control” principle to ingrown toenail management, suggesting that immediate surgical intervention may represent a feasible and clinically reasonable strategy without an unacceptable increase in complication risks. However, formal non-inferiority trials would be needed to definitively confirm equivalent safety profiles.

This study has several limitations that warrant consideration. First, its retrospective nature may have introduced inherent biases in data collection and documentation. Second, we did not employ validated objective clinical scoring systems, such as the Foot and Ankle Outcome Score or the Foot and Ankle Ability Measure [20], which limits our ability to quantitatively assess functional improvement and patient-reported quality of life beyond satisfaction and recurrence rates. Third, while our overall sample size was large, the number of cases in Group 1 was relatively small (*n* = 76) compared to Group 2, which may affect the statistical power to detect subtle differences in complications. Fourth, a significant portion of the initial cohort was evaluated via telephone recall (179 toes, 46.1%) rather than direct chart review (209 toes, 53.9%), and 78 toes (16.7%) were lost to follow-up, which potentially introduces attrition bias. This loss to follow-up could affect outcome estimates in opposite ways: asymptomatic patients with successful outcomes might have lacked incentive to respond to phone recalls, whereas patients experiencing late complications or dissatisfaction might have sought care at secondary clinics and been uncontactable. Nevertheless, the overall loss rate of 16.7% remains within a reasonable range for long-term retrospective study designs. Furthermore, our sensitivity analysis restricted to the sub-cohort evaluated exclusively through in-person chart reviews (*n* = 209) demonstrated virtually identical comparative outcomes between groups, confirming that loss to follow-up is unlikely to have systematically biased our primary conclusions regarding the safety and efficacy of the immediate Winograd procedure. Furthermore, outcome ascertainment differed between direct chart review (*n* = 209) and telephone recall (*n* = 179). Because telephone assessments relied on patient self-reports, minor asymptomatic recurrences or mild, self-limiting postoperative surgical site infections might have been underreported compared to objective in-person clinical examinations. To mitigate this information bias, telephone interviews were strictly conducted using a standardized protocol focusing on concrete clinical endpoints (e.g., visible nail spicule regrowth, purulent discharge, and extended antibiotic prescriptions). Crucially, our sensitivity analysis restricted to patients evaluated solely via objective chart reviews (*n* = 209) yielded findings identical to those of the overall cohort, confirming that the mode of outcome assessment did not alter our primary conclusion that chronic severe preoperative infection does not adversely affect outcomes of the Winograd procedure. Fifth, formal interobserver agreement metrics (such as Cohen’s kappa) and prospective assessor calibration were not evaluated for outcome determination, although data extraction was performed independently using standardized operational criteria and consensus resolution. Finally, the diagnosis of chronic severe preoperative local infection was based on clinical signs rather than standardized laboratory or microbiological markers, which may lead to variability in classification. Further prospective, multi-center trials using standardized functional scores and objective inflammatory markers are needed to validate these findings. Finally, the diagnosis of chronic severe preoperative local infection was based on clinical signs rather than standardized laboratory or microbiological markers, which may lead to variability in classification. Regarding generalizability, while the Winograd procedure and oral cephalosporin therapy are widely practiced clinical standards globally, all operations in this study were conducted at a single tertiary center by fellowship-trained foot and ankle specialists utilizing meticulous matricectomy, electrocautery, and thorough debridement. Consequently, these favorable outcomes may not automatically generalize to primary care settings or non-specialized surgical practices. Further prospective, multi-center trials using standardized functional scores and objective inflammatory markers are needed to validate these findings across broader clinical settings.

## 5. Conclusions

Our study demonstrated that the Winograd procedure is a highly feasible and effective surgical intervention for the treatment of ingrown toenails of the great toe, achieving comparable clinical outcomes without a statistically significant increase in major postoperative complications or recurrence in the presence of chronic severe preoperative local infection. Our findings underscore that radical and immediate excision of the offending nail spicule, alongside the thorough debridement of inflammatory granulation tissue and the germinal matrix, allows for direct source removal and favorable healing for severely infected cases. Furthermore, we identified that while patients presenting with chronic severe local infection tend to be younger and have a higher prevalence of smoking, these factors do not preclude a successful clinical outcome when a meticulous surgical technique is employed. While a finding of no statistically significant difference does not inherently establish equivalent safety, these observational data suggest that an immediate Winograd procedure can be considered a reasonable primary treatment option when accompanied by thorough debridement. Nevertheless, a thorough understanding of the matricectomy process and the elimination of dead space are essential to optimize long-term clinical success and symptomatic improvement.

## Figures and Tables

**Table 1:** Baseline characteristics of overall great toes (*n* = 388) and comparison according to the presence of preoperative signs of chronic severe local infection.

Parameters	Overall (*n* = 388)	Group 1 (Chronic Severe Preoperative Local Infection, *n* = 76)	Group 2 (No or Mild Preoperative Local Infection, *n* = 312)	*p*-Value
Age at the time of surgery (years)	37.6 ± 20.3	29.7 ± 18.5	39.5 ± 22.7	0.001
Women	149 (38.4%)	24 (31.6%)	125 (40.1%)	0.173
Smoking at the time of surgery (yes)	25 (6.4%)	9 (11.8%)	16 (5.1%)	0.033
Past medical history	Hypertension 39 (10.2%) Diabetes 34 (8.9%) Coronary heart disease 12 (3.2%) ESRD 7 (1.9%) Hyperthyroidism 5 (1.3%) Hypothyroidism 4 (1.0%) Crohn’s disease 2 (0.6%)	Hypertension 5 (6.6%) Diabetes 3 (3.9%) Coronary heart disease 1 (1.3%) ESRD 1 (1.3%) Hypothyroidism 2 (2.6%)	Hypertension 34 (10.9%) Diabetes 31 (9.9%) Coronary heart disease 11 (3.5%) ESRD 6 (1.9%) Hyperthyroidism 5 (1.6%) Hypothyroidism 2 (0.6%) Crohn’s disease 2 (0.6%)	0.262 (hypertension) 0.098 (diabetes) 0.474 (coronary heart disease) 1.000 (ESRD) 0.174 (hypothyroidism)
Affected side (Right or left)	Right 191 (49.2%) Left 197 (50.8%)	Right 38 (50.0%) Left 38 (50.0%)	Right 153 (49.0%) Left 159 (51.0%)	0.88
Affected location (Medial or lateral)	Medial only 128 (32.8%) Lateral only 154 (39.9%) Both 106 (27.3%)	Medial only 12 (15.8%) Lateral only 37 (48.7%) Both medial and lateral 27 (35.5%)	Medial only 116 (37.2%) Lateral only 117 (37.5%) Both medial and lateral 79 (25.3%)	0.073 (both medial and lateral)
Follow-up duration (months)	34.8 ± 6.9 (range, 6–97)	32.4 ± 7.5 (range, 6–97)	35.4 ± 6.5 (range, 6–96)	0.125

Data presented as mean ± SD or as *n* (%) and *p*-values represent comparisons between Group 1 and 2. ESRD, end-stage renal disease.

**Table 2:** Comparison of postoperative clinical course and complications according to the presence of preoperative signs of chronic severe local infection at the affected site (*n* = 388 great toes).

Parameters	Group 1 (Chronic Severe Preoperative Local Infection, *n* = 76)	Group 2 (No or Mild Preoperative Local Infection, *n* = 312)	*p*-Value
Mean duration of oral antibiotics after the surgery (days)	13.9 ± 7.5 (range, 5–37)	10.6 ± 7.8 (range, 5–46)	0.114
Superficial surgical site infection	24 (31.6%)	88 (28.2%)	0.561
Persistent surgical site infection lasting more than 3 weeks	12 (15.8%)	36 (11.5%)	0.313
Mean duration of antibiotic use in cases of persistent surgical site infection lasting more than 3 weeks (days)	33.0 ± 3.4 (range, 26–37)	36.6 ± 5.6 (range, 28–46)	0.094
Secondary surgical intervention due to a failure of infection eradication	0	0	N/A
Postoperative recurrence	10 (13.2%)	48 (15.3%)	0.625

Data presented as mean ± SD or as *n* (%).

**Table 3:** Multivariable logistic regression analysis for postoperative complications and recurrence.

Outcome/Predictor	Adjusted Odds Ratio	95% Confidence Interval	*p*-Value
Superficial Surgical Site Infection			
Group 1 (vs. Group 2)	1.15	0.68–1.95	0.582
Age (per year increase)	0.99	0.98–1.01	0.420
Smoking (Yes vs. No)	1.42	0.55–3.65	0.452
Persistent Surgical Site Infection			
Group 1 (vs. Group 2)	1.32	0.62–2.80	0.461
Age (per year increase)	1.00	0.98–1.02	0.912
Smoking (Yes vs. No)	1.55	0.48–4.98	0.455
Postoperative Recurrence			
Group 1 (vs. Group 2)	0.88	0.42–1.85	0.742
Age (per year increase)	1.01	0.99–1.03	0.315
Smoking (Yes vs. No)	1.20	0.35–4.12	0.771

## Data Availability

The data that support the findings of this study are available from the corresponding author [J.Y. Choi], upon reasonable request.

## Abbreviations

The following abbreviations are used in this manuscript:

aOR	Adjusted odds ratio
CI	Confidence interval
